# Three dimensional condylar positional and morphological changes following mandibular reconstruction based on CBCT analysis: a prospective study

**DOI:** 10.1186/s13005-023-00347-4

**Published:** 2023-02-07

**Authors:** Saddam Noman Al-Wesabi, Bassam Abotaleb, Eissa Abdo Al-Shujaa, Abdo Ahmed Mohamed, Khaled Alkebsi, Wael Telha, Sun Jian, Xie Fuqiang

**Affiliations:** 1 Lanzhou University, Second Hospital, Oral and Maxillofacial Surgery Department, Lanzhou, Gansu Province China; 2 State Key Laboratory of Oral Diseases and National Clinical Research Centre for Oral Diseases, Department of Oral implantology, West China Hospital of Stomatology, Sichuan University, Chengdu, Sichuan China; 3 Department of Oral and Maxillofacial Surgery, Faculty of Dentistry, Ibb University, Ibb, Yemen; 4 Department of Oral & Maxillofacial Surgery, Sun Yat-sen Memorial Hospital, Sun Yat-sen University, Guangzhou, China; 5 State Key Laboratory of Oral Diseases and National Clinical Research Centre for Oral Diseases, Department of Oral and Maxillofacial Surgery, West China Hospital of Stomatology, Sichuan University, Chengdu, Sichuan China

**Keywords:** Three-dimensional analysis, Reconstruction with condylar preservation, Cone-beam computed tomography (CBCT), Mandibular reconstruction, Free fibula flap

## Abstract

**Objective:**

This study aimed to evaluate the condylar positional changes following mandibular reconstruction with preservation of the condylar head using Cone-Beam Computed Tomography (CBCT). Also, to assess joint space changes and the overall volumetric space compared to the preoperative status.

**Methodology:**

This prospective study included 30 patients (60 joints) subjected to unilateral mandibular resection and reconstruction with preservation of the condylar head. The Helkimo index and preoperative (T1), two weeks postoperative (T2), and follow-up CBCTs (T3) after at least six months were gathered and processed to evaluate the condylar position and TMJ joint space using Anatomage Invivo 6. A student’s t-test and repeated-measures ANOVA statistics were used. A *P* value of less than 0.05 was considered statistically significant.

**Results:**

Thirty patients (14 males, 16 females) with a mean age of 40.01 ± 12.7 years (a range of 18.1–62.9 years) were included. On the tumor side, there were significant variances in the vertical and mediolateral condylar positions between the three-time points (T1, T2, T3). Immediately after the operation, the condyles were significantly displaced in a downward direction at T2, which became larger after the last follow-up period (T3) (*p* = 0.007). The condylar positions at the anteroposterior direction were relatively stable without significant differences between the three times points (*p* = 0.915). On the non-tumor side, the condylar positions were relatively stable in the mediolateral and anteroposterior positions.

In the tumor side, all of the TMJ spaces were significantly increased in size following the mandibular reconstructions (T2 and T3). However, on the non-tumor side, the anterior, posterior, and medial joint spaces were significantly changed postoperatively.

**Conclusion:**

After mandibular reconstruction with condylar preservation, the condylar position and volumetric measurement immediately changed noticeably and continued to be a permanent change over time compared to relatively stable condyles on the non-tumor side. According to Helkimo index, patients become adapted to the postoperative changes without significant differences between the two sides.

**Supplementary Information:**

The online version contains supplementary material available at 10.1186/s13005-023-00347-4.

## Introduction

Reconstruction of oral and maxillofacial defects is most common following extirpative surgery for malignant or benign diseases [1–3]. Mandibular reconstruction using the free fibula flap remains the gold standard method for patients undergoing mandibular resection in cancer, trauma, osteoradionecrosis, and infection [4, 5]. The goals of mandibular reconstruction are repairing the essential structural, functional recovery of the mandible and improving the esthetic status of the orofacial complex. In addition, it contributes to normalizing speech, cosmesis, and deglutition [6, 7]. Furthermore, it has a significant effect on the psychological condition of patients, improving their quality of life [8].

The function of the TMJ could be preserved after hemimandibulectomy without resection of the condylar head [6]. However, further investigation may be required to provide a clear view of the outcomes of such surgical procedures. Ricketts [9] introduced joint space measurements using the appropriate measures of radiographic joint spaces between the mandibular condyle and the glenoid fossa and evaluated the mandibular condyle and the glenoid fossa. However, previous studies on mandibular microvascular reconstruction have primarily focused on the operational outcome and relation of the bony construct, whereas changes in condylar position have only been scantly investigated [10].

Our study aimed to investigate the effects of mandibular reconstruction on three-dimensional changes in the condylar position, TMJ volume, and TMJ space following hemimandibular resection and reconstruction with preservation of the affected side condyle. The investigation was performed based on the hypothesis that after mandibular reconstruction with condylar preservation and reconstruction, no significant changes will be found between the preoperative and postoperative parameters and between the tumor and the non-tumor side.

## Materials and methods

### Study design

The study was designed as a prospective study including patients who underwent a unilateral mandibular reconstruction with preservation of the condylar head at Lanzhou University’s Second Hospital’s maxillofacial surgery department between October 1, 2019, and March 1,2022, However, the last patients were included in September 1, 2021, with a follow-up period of at least six months. The study was approved by the ethical committee of the Stomatology School of Lanzhou University with the approval No (LZUKQ-2019-047). The study followed the guidelines of the Declaration of Helsinki.

### Criteria of study

The inclusion criteria were as follows: consecutive patients above the age of 18 with a unilateral mandibular tumor, mandibular reconstruction with condylar head preservation, patients who have CBCT in all follow-up steps with a follow-up after at least six months, and no history of previous TMJ surgery. Patients with a bilateral reconstruction, early recurrence of malignancy, immediate flap failure due to venous thrombosis, and patients without CBCT were excluded.

### Sample size

The sample size was calculated using G. power analysis software (University of Dusseldorf, Dusseldorf, Germany) at a significant level equal to 0.05, power equal to 99%, and an effect size of differences in the measure of Superior joint space at three-time intervals as reported in a previous study [11]. The power analysis revealed a need for 30 subjects to be enrolled in the present study.

### CBCT analysis

**Fig. 1:**
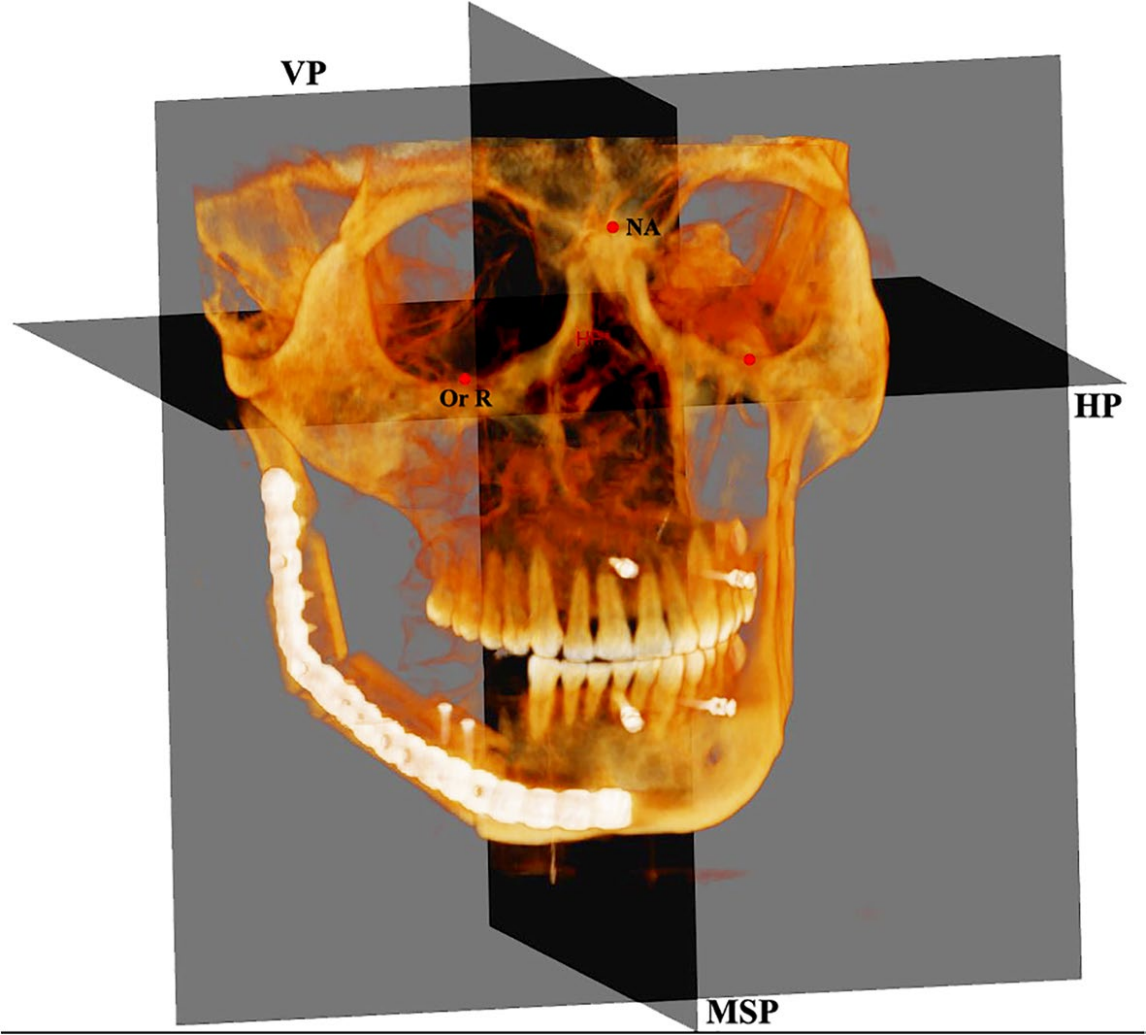
Three planes. Three planes. MSP: midsagittal plane, VP: vertical plane, HP: horizontal plane

**Fig. 2:**
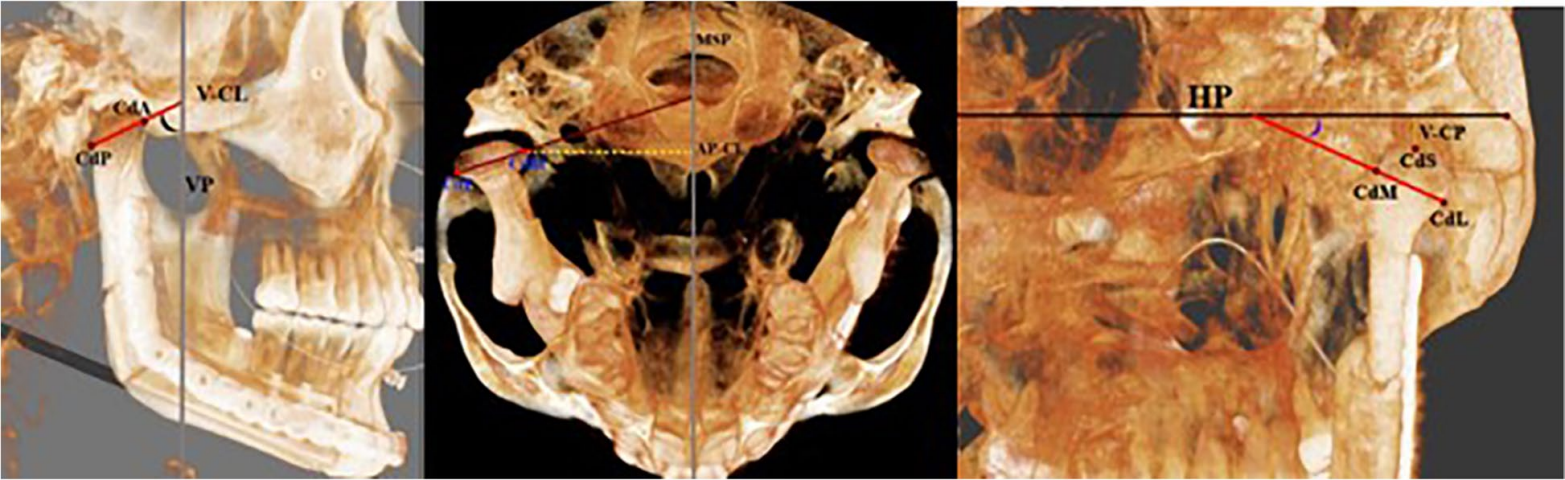
**A** Condyle angulation and position in the vertical plane (VP), CdA: anterior condylar point, CdP: posterior condylar point, V-CL: vertical condylar inclination; **B** Condyle position and angulation in the midsagittal plane (MSP). AP-CL Anteroposterior condylar inclination, CdM: medial condylar point, CdL: lateral condylar point, CdS: superior condylar point, C-VP: vertical condylar position, **C** Position, and angulation to the horizontal plane (HP), CdM: medial condylar point, CdL: lateral condylar point, CdS: superior condylar point

CBCT one week before surgery T1, two weeks after surgery T2, then after at least six months T3. All CBCTs and related data forms were collected, and slices were adjusted to obtain the same value as the CBCT image. The Cone Beam Computed Tomography analysis was achieved from Cone Beam CBCT images by New Tom VGI Imaging System (QR R Italy); all Subjects were scanned using a standard protocol which included a 16.0 cm x 16.0 cm field of view, standardized head position, maximum teeth intercuspation, the horizontal plane (HP) parallel to the floor, exposure parameter settings (tube voltage =110 kV, filament current = 29.43 mAs, total scan time = 1.8 s), and image acquisition at 0.3 mm voxel size. The TMJ images were analyzed in 3D using the Invivo Anatomage version 6 software (Anatomage, San Jose, CA, USA). To assess the mandibular condylar position relative to the cranial base, we evaluated according to the relationship of the condyle with the horizontal plane, vertical plane, and mid-sagittal plane. TMJ space measures were defined by selecting TMJ points and analyzed as a 3D mold based on three planes: coronal, axial, and sagittal (X, Y, and Z). Tables 1, 2, 3 and 4 show 3D craniofacial skeletal landmarks, reference planes and lines temporomandibular landmarks, 3D measurements of the condylar position, and three-dimensional measurements of the mandibular condyle and TMJ spaces. Figure 1 shows the cranial-facial skeleton reference planes and lines. Figure 2 demonstrates measurements of the condylar position on the tumor and non-tumor sides.

**Table 1:** The craniofacial skeletal landmarks used in the study

Landmark	Abb.	Definition
Nasion	N	The anterior and superior frontonasal sutures meet in the middle.
Sella	S	The hypophyseal fossa’s centre is in the middle cranial fossa (Sella turcica).
Basion	Ba	The cranial base’s posterior tip. Sagittally, the foramen magnum’s most inferior posterior point.
Orbital	Or	The lowest point on the orbit’s inferior border is left or right.
Right-Left Porion	Po	The external auditory meatus’s most outer and superior bony points are right and left.
Gonion	Go	The place where the ramus line and the mandible body line connect at a bisecting angle.
Gnathion	Gn	The mandibular symphysis’s most anterior and inferior point on the contour.
Anterior nasal spine	ANS	The most anterior and middle of the maxilla’s anterior nasal spine
IncisiveForamen	IF	The incisive foramen centre was mediolaterally at the maxillary mid palatine and existed posterior to the central incisors.
Posterior nasal spine	PNS	The palatine bone’s most posterior midpoint of the posterior nasal spine

**Table 2:** Reference planes and lines of temporomandibular landmarks

Reference Plane / line		Abb.	Definition
Reference Plane	Horizontal Plane	HP	It’s composed of a three-point right Orbitale and two sides of porion.
	Mid-Sagittal Plane	MSP	Sella and Nasion created a perpendicular plane to the horizontal plane.
	Vertical Plane	VP	The plane passes through the sella and is perpendicular to the sagittal and horizontal planes.
Reference line	Mandibular fossa line	MFL	The two bony mandibular fossa MF points are used to draw a line.
	TM line	TML	A line is created by the point of the Anterior Tubercle and the tip of the Auditory Meatus.
	Sagittal condyle neckline	CNL	A line extends from ACN–PCN.
	Anteroposterior condylar line	CdA - CdP	A line extends from CdA to CdP.
	Mediolateral condylar line	CdM - CdL	A line extends from CdM to CdL.

**Table 3:** 3D measurements of condylar position, inclination, and TMJ space

Point’s name	Abb.	Definition
Medial joint space “Tubercle point.”	JMS-f	The most right or left lateral point of the mandibular fossa’s medial wall.
Medial Condylar point	CdM	The most right or left medial point on the condylar head.
Articular tubercle	AT	The articular tubercle’s most inferior and posterior points.
Inferior meatus	IM	The point on the right or left external auditory meatus is the most inferior and lateral.
Anterior joint space “Mandibular fossa point”	AJS1	The smallest distance between the most posterior point of the mandibular fossa’s right or left anterior wall and the anterior condyle fossa’s shortest distance.
Anterior joint space “Condylar point.”	AJS2	The shortest distance between the most anterior point of the right or left condyle and the fossa is opposite the most anterior point of the right or left condyle.
Posterior joint space “Mandibular fossa point”	PJS1	The shortest distance between the most anterior point of the mandibular fossa’s right or left posterior wall and the most posterior point of the posterior condyle fossa.
Posterior joint space “Condylar point.”	PJS2	The most posterior point of the right or left condyle is directly opposite the shortest posterior condyle fossa distance.

**Table 4:** Three dimensional measurements of mandibular condyle and TMJ spaces

	No	Measurement	Abb	Definition
Mandibular condyle position	1	Vertical point condylar position	V-CP	The vertical distance between the CdS and HP
	2	Anteroposterior condylar point position	AP-CP	The anteroposterior distance between the CdA and VP
	3	Mediolateral condylar point position	ML-CP	The mediolateral distance between the CdA and MSP
TMJ space parameters	1	Anterior Joint Space	AJS	The shortest distance between AJS1 and AJS2
	2	Superior Joint Space	SJS	The shortest distance between SJS1 and SJS2
	3	Posterior Joint Space	PJS	The shortest distance between PJS1 and PJS2
	4	Medial Joint Space	MJS	The shortest distance between MJS-F and CdM
	5	Vertical condylar joint position	V- CJP	The linear difference between condylar height to TM line and mandibular fossa height to TM line

**Fig. 3:**
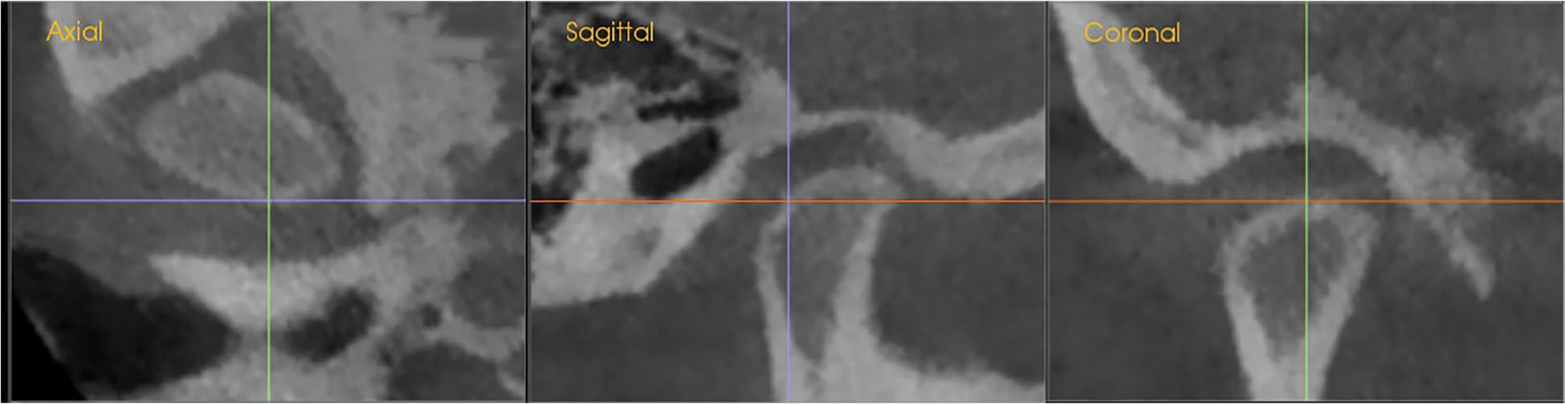
Three planes slice locator

The orientation of the coordination system was set based on the points of facial skeletal midline: Nasion, incisive foramen, and basion, confirmed by Garcia et al. as correct sites and validated by the previous studies with different objectives [12–14]. The lateral landmarks were determined by the right orbital (Or) point and two porion (Pr) points; second, the following landmarks were adjusted to the exact position of the selected points and digitized separately using three planes slice locator (sagittal, coronal, and axial) Fig. 3.

**Fig. 4:**
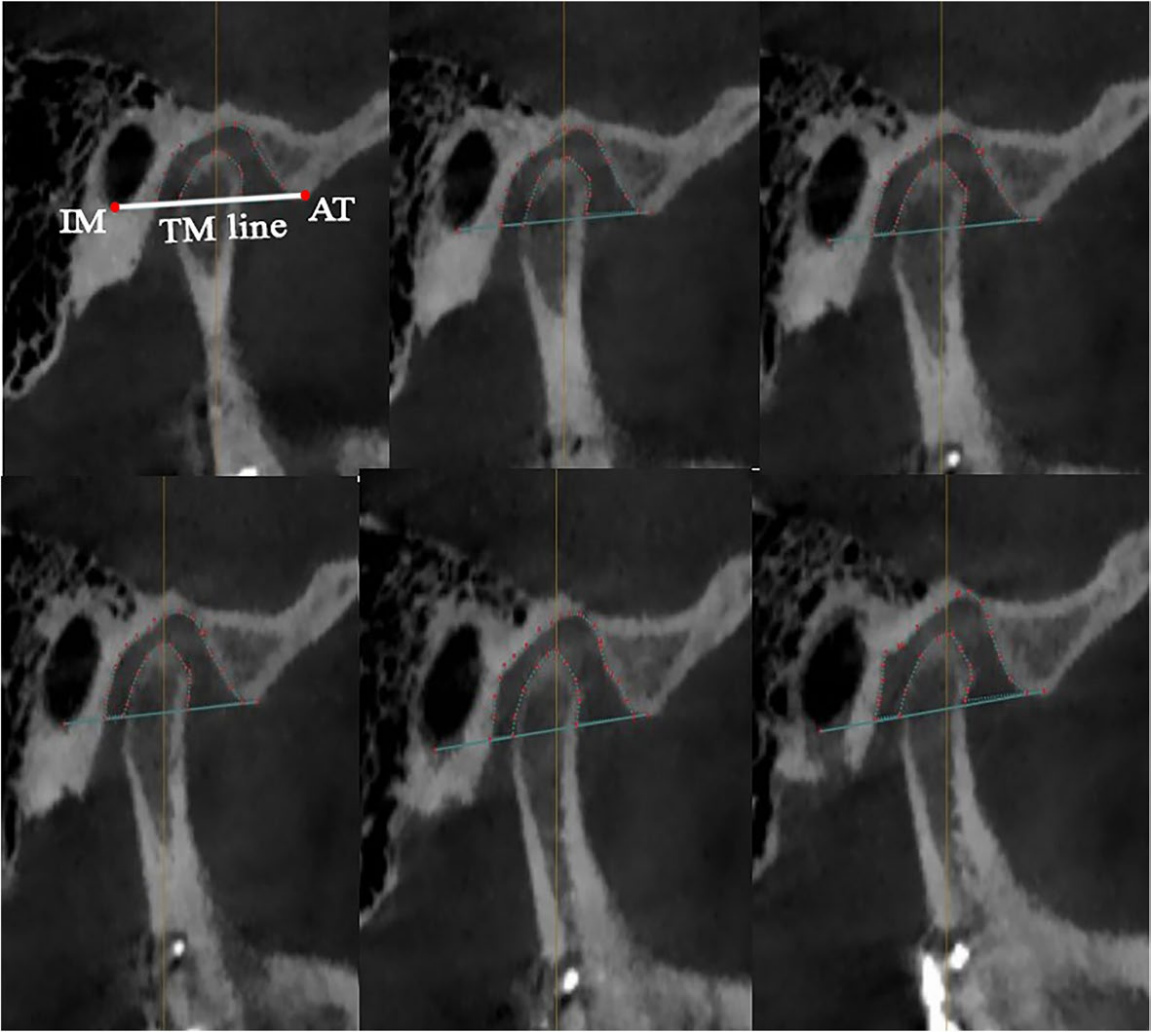
Three-dimensional Volumetric measurement of the TMJ spacel

Volumetric joint space: A TM line was drawn from the inferior point of the auditory meatus (IM) to the inferior point of the articular tubercle (AT) to determine the inferior border of the whole joint space Fig. 4, To measure the whole joint space we used the cubic three-dimensional analysis of volumetric joint space by sectioning the whole joint space into six sections, where every section had a thickness of 1.5 mm and a width of 80 mm at an interval of 0.75 mm, and then calculated the space with the equation of sigma volume:

$$v\cong {\Sigma}_{k=1} A\left({x}_{\dot{I}}\right)\Delta x$$

To further evaluate the reproducibility of the results, a random sample of 30% of the total analyzed sample was measured twice at three-week intervals by the same assessor (S.N).

### Clinical assessment

Gender, age, primary location, pathology, and defect type were all recorded for each patient. For the clinical outcome, the study used the Helkimo index scoring system, which accurately assesses the TMJ function [15, 16]. Helkimo Ai represents subjective symptoms of TMJ dysfunction, whereas the Di index evaluates the TMJ dysfunction by clinical assessment of TMJ pain, impaired TMJ function, muscular pains, and reduced mouth movement.

In this study, the Ai was obtained and divided into three categories: Ai 0, Ai I, and Ai II. Ai 0 indicates no symptoms are present. Ai I denotes moderate symptoms with at least one of the following: stiffness during exercise, muscle fatigue, and stiffness in the morning or during exercise. Ai II indicates severe symptoms, including at minimum one of the following: joint noise, locking of the joint, mouth opening restriction, dislocation, mandibular pain, and masticatory muscle pain or TMJ caused by motion. For objective clinical assessment (Di), joint dysfunction, mouth discomfort, limited mandible movement, and muscular soreness were used. Every feature was given a score ranging from 0 to 5 on a scale of 1 to 5. The total score for each feature was as follows: 0 = Di 0 (normal function), 1–4 = Di I, 5–9 = Di II, and 10–30 = Di III (worst function) [17].

### Surgical technique

Two highly qualified surgeons with over twenty years of experience treated all reconstruction cases. A two-team procedure was used, the head and neck team performed the mandibular ostectomy, in view of surgical margin and subsequent fixation, and preserved the condylar head in the glenoid fossa, and reproduced the original occlusion and condylar position after flap positioning. The other team used the approach to harvesting the fibula flaps. All flaps were osteotomized according to the defect size and according to the design of virtual surgical planning using a cutting guide template.

For contouring. Intermaxillary fixation was used for occlusion (IMF). The condylar head and fibula were fixed in place using reconstruction plates. After that, microsurgical vascular anastomosis was performed. After resection and reconstruction of the defect, we performed IMF for two weeks, then removed and made the second CBCT T2.

### Statistical analysis

The Statistical Package for Social Sciences (IBM SPSS Statistics) software, version 25 (IBM Corp.) for Windows, was used to analyze the data. This study includes 30 patients, which means we have 60 joints (30 joints on the Tumor sides and 30 joints non-Tumor side). The significance level was set at *P* < 0.05. Shapiro-Wilk test was used to check for normal distribution. The repeated-measures analysis of variance (ANOVA) was conducted to check the presence of different time-dependent changes (T1–T2, T1–T3, and T2–T3) of the condylar positional change within the tumor side and non-tumor side groups, as well as in between them.

To evaluate reproducibility and reliability, researchers used intra-class correlation coefficients (ICCs) and absolute and relative technical error of measurement tests (the absolute technical error of measurement [TEM], relative TEM [rTEM], and a coefficient of reliability [R]). The student’s t-test was used to statistically analyze data between tumor and non-tumor sides. It was carried out using GraphPad Prism 9, which efficiently performs basic statistical tests commonly used by laboratory and clinical researchers.

## Results

### Demographics data

Among 59 patients, 30 patients with condylar head preservation were included in this study (14 males and 16 females), with a mean age of 40.01 ± 12.7 years (range 18.1–62.9 years). Squamous cell carcinoma was the most common reason for mandibular resection (36.67%), followed by ameloblastoma (30%), keratocystic odontogenic tumor (13.33%), dentinogenic ghost cell tumor (10%), and osteoblastoma (6.67%). The follow-up ranged from 6 to 13 months, with a mean of 6.97 and 1.2 months. The intra-observer reliability was excellent, above 0.95 (Table 5).

**Table 5:** Inter and Intra-observer TEM, rTEM, and R of the measurements and Cronbach alpha test through the Intraclass correlation coefficient (ICC)

Measurements		Intra-Observer Error			
		ICC	TEM	r TEM	R^**a**^
1	G-ICD	0.995	0.6040	0.6257	0.9903
2	M-ICD	0.997	0.6990	0.8538	0.9945
3	L-ICD	0.996	0.6442	0.5776	0.9921
4	ML-CI-R	0.989	0.6131	10.0720	0.9786
5	ML-CI-L	0.990	0.7135	11.0627	0.9823
6	V-CP-L	.992	0.3421	13.6680	0.9760
7	V-CP-R	0.987	0.3107	11.9714	0.9696
8	AJS-R	0.978	0.4045	10.0426	0.9555
9	PJS-R	0.972	0.2869	6.3932	0.9508
10	MJS-R	0.978	0.3662	9.1665	0.9551
11	SJS.R	0.980	0.2535	5.5668	0.9627
12	AJS-L	0.980	0.3173	9.5101	0.9627
13	PJS-L	0.982	0.2050	5.5404	0.9669
14	MJS-L	0.979	0.2827	7.3319	0.9623
15	SJS.L	0.983	0.2317	5.4595	0.9698

*TEM* Absolute technical error of measurements

*rTEM* Relative technical error of measurements

^a^R is the coefficient of reliability. *ICC* Intraclass correlation coefficient

### Condyle position

Regarding the tumor side, there were significant variances in the vertical and mediolateral condylar position between the three-time points (T1, T2, T3). Immediately after the operation, the condyles were significantly displaced in the downward direction at T2, which became larger after the last follow-up period (T3) (*p* = 0.007). Likewise, the condyles were significantly displaced in the lateral direction at T2, which increased at T3 (*p* = 0.003). The condylar position at the anteroposterior direction was relatively stable without significant differences between the three times points (*p* = 0.915) (Table 6).

**Table 6:** Descriptive statistics and significant (*P*) values of analysis of variance (Repeated measures analysis of variance (ANOVA),) for the Condylar position

Measurements		Side	T1 Mean and Std. Deviation	T2 Mean and Std. Deviation	T3 Mean and Std. Deviation	(*P*) values
Condylar position	ML-CP (MSP)	Tumor side	53.21 ± 6.71 mm	55.46 ± 5.52 mm	56.39 ± 5.16 mm	0.003
		Non-Tumor side	56.04 ± 5.97 mm	56.08 ± 5.17 mm	55.67 ± 5.66 mm	0.845
	V-CP (HP)	Tumor side	2.90 ± 1.48 mm	3.01 ± 1.95 mm	3.48 ± 1.89 mm	0.007
		Non-Tumor side	2.62 ± 1.88 mm	2.27 ± 1.47 mm	2.59 ± 1.63 mm	0.042
	AP-CP (VP)	Tumor side	5.30 ± 3.42 mm	5.62 ± 3.06 mm	5.50 ± 2.59 mm	0.915
		Non-Tumor side	5.84 ± 3.29 mm	4.51 ± 3.36 mm	5.31 ± 3.03 mm	0.275

*ML-CP* Mediolateral Condylar position, *V-CP* Vertical Condylar position, *AP-CP* Anteroposterior Condylar position

On the non-tumor side, the condylar positions were relatively stable in the mediolateral and anteroposterior positions (*p* = 0.845 and 0.275, respectively) (i.e., no significant differences between measurements of the three time points). However, there were significant variances found in the vertical position of the condyles among the three time points (*p* = 0.042). The condyles were immediately displaced inferiorly (T2), which became largely displaced at T3 Table 6.

### TMJ spaces

**Fig. 5:**
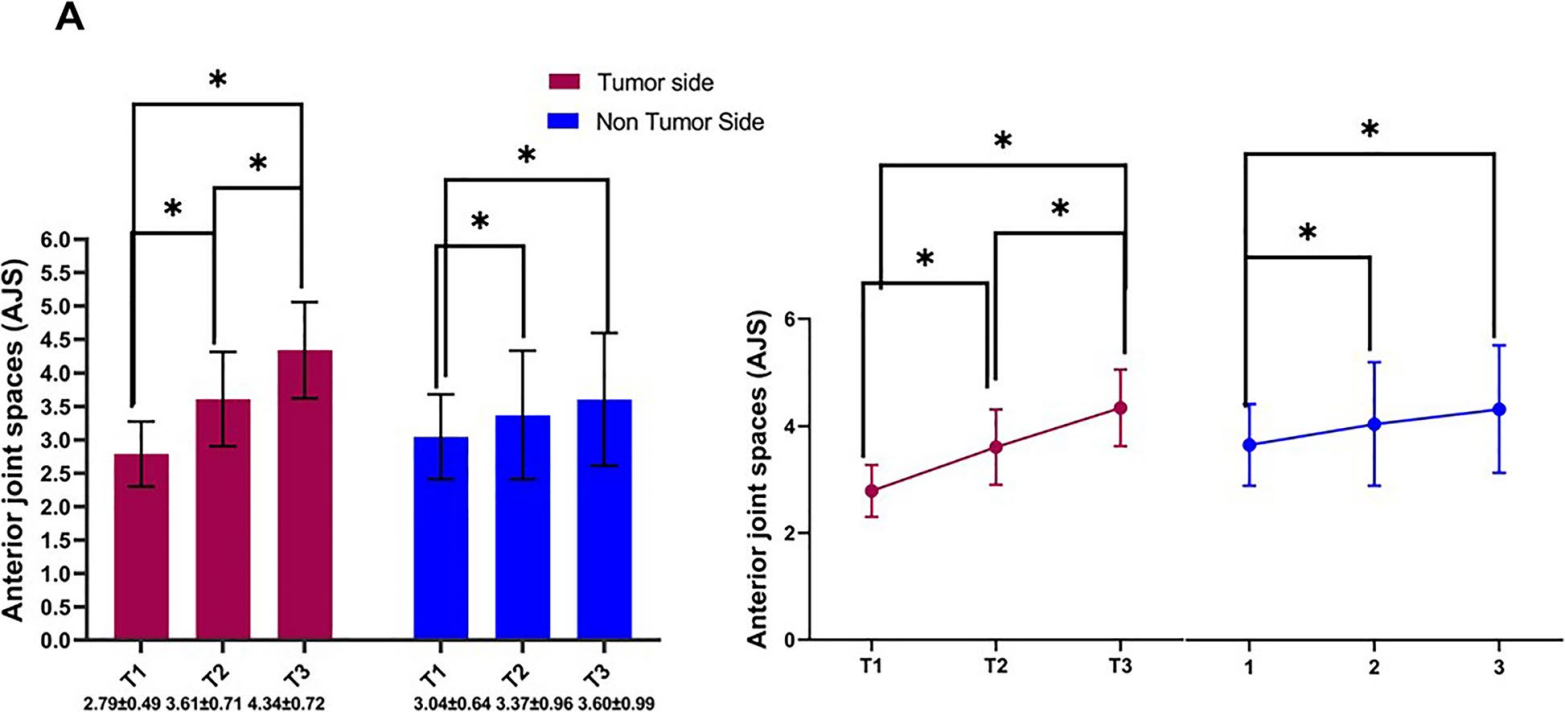
Significant (*P*) values of analysis of variance TMJ spaces anterior. **A** Anterior joint space. T1 before surgery, T2 after surgery, T3 after surgery follow-up

In the tumor side, all the TMJ spaces were significantly increased in size following the mandibular reconstructions (T2 and T3) (Tables 6 and 7, Fig. 5). However, on the non-tumor side, the anterior, posterior, and medial joint spaces were significantly changed postoperatively. The superior joint spaces were increased at T2 and T3, but this change was not statistically significant Tables 6, 7, and Fig. 5.

**Table 7:** Descriptive statistics and significant (*P*) values of analysis of variance (Repeated measures analysis of variance (ANOVA)) for the TMJ Spaces

Measurements		Side	T1 Mean and Std. Deviation	T2 Mean and Std. Deviation	T3 Mean and Std. Deviation	(***P***) values
**TMJ Spaces**	AJS	Tumor side	2.79 ± 0.49 mm	3.61 ± 0.71 mm	4.34 ± 0.72 mm	0.000
		Non-Tumor side	3.04 ± 0.64 mm	3.37 ± 0.96 mm	3.60 ± 0.99 mm	0.000
	SJS	Tumor side	4.35 ± 0.82 mm	5.09 ± 1.21 mm	4.64 ± 0.90 mm	0.003
		Non-Tumor side	4.74 ± 1.36 mm	4.71 ± 1.23 mm	4.78 ± 1.22 mm	0.814
	PJS	Tumor side	3.65 ± 1.14 mm	4.07 ± 1.44 mm	4.64 ± 1.34 mm	0.001
		Non-Tumor side	3.23 ± 0.79 mm	3.48 ± 0.90 mm	3.69 ± 0.78 mm	0.045
	MJS	Tumor side	3.91 ± 1.02 mm	5.18 ± 1.81 mm	5.50 ± 2.49 mm	0.000
		Non-Tumor side	3.71 ± 1.16 mm	4.17 ± 1.57 mm	5.07 ± 1.40 mm	0.001
	VTJS	Tumor side	65.77 ± 13.41 mm3	74.10 ± 20.15 mm3	72.72 ± 15.48 mm3	0.006
		Non-Tumor side	69.47 ± 13.70 mm3	69.64 ± 16.08 mm3	68.97 ± 11.81 mm3	0.856

### Volumetric joint space

Repeated-measures analysis of variance (ANOVA) showed a statistical difference in volumetric joint space among the three time point measurements T1, T2, and T3 on the tumor side (*p* = 0.006). The volumetric space was significantly increased at T2 (74.10 ± 20.15 mm3),  which did not return to the normal volume at T3 (72.72 ± 15.48 mm3). However, the volumetric joint space in non-tumor side remained stable without significant changes postoperatively (*p* = 0.856) Tables 6 and 7.

### Helkimo index for patients

Out of 30 patients, 25 patients were assessed by the Helkimo index as follows: Ai 0 was reported in 15 patients (highest), Ai I was found in 9 patients, and Ai II was found in 1 patient. Regarding the objective clinical evaluation, Di 0 was found in 4 patients, Di I was reported in 13 patients, and eight patients were assessed as Di II. In contrast, none of the patients were reported with Di III. Moreover, five patients dropped out from the Helkimo index assessment (3 patients were lost during follow-up, and two were not cooperating) Table 8.

**Table 8:** Helkimo index result for tumor and non-tumor sides

Helkimo index score for patients who have been following up for more than six months	Tumor side	Non -tumor side
Di		
DiO	4	17
DiI	13	6
DiII	8	2
DiIII	0	0
Ai		
AiO	15	19
AiI	9	6
AiII	1	0

In order to detect the correlation between the clinical finding and the condyle position and joint space, the Pearson’s correlation was conducted and revealed a non-significant relationship between the Helkimo index result and the other variables at T3 (*P* > 0.05), which indicates that the condyle position has an excellent adaptive position Table 9.

**Table 9:** CBCT Measurement with Helkimo index correlation

	Ai Helkimo		Di Helkimo	
	Pearson’s correlation	*P* value	Pearson’s correlation	*P* value
MLCPT3	0.249	0.23	0.34	0.08
AP_CpT3	−0.146	0.48	−0.255	0.21
V_CP_T3	0.087	0.681	0.119	0.572
AJS_T3	0.186	0.373	−0.026	0.901
PJS_T3	−0.141	0.500	0.007	0.972
MJS_T3	−0.059	0.778	−0.086	0.684
SJS_T3	−0.022	0.918	−0.039	0.852

## Discussion

Reconstruction of oral and maxillofacial defects is the most common treatment after extirpative surgery for malignant or benign diseases [18]. TMJ dysfunction is a common complication of condylar head reconstruction following tumor removal of the hemimandible. After hemimandibulectomy without resection of the condylar head, the TMJ function is preserved [6]. The limitations of mandibular reconstruction are associated with TMJ dysfunction. Because the previous studies did not record the TMJ function as the clinical outcome, it was affected by many factors, such as the small patient enrollment number in the previous study, radiographic technique, and the accuracy of the clinical examination of the condyle position and TMJ space. Our study focuses on the method accuracy, clinical outcome, and the sample size of patients enrolled in this study [11].

Anatomic studies have shown that the condylar head has adequate vascularity. In contrast, histological studies examining tumor transmission patterns have demonstrated that the condyle is rarely affected in malignancies that originate inside the body and ramus [19, 20]. The condyle can be preserved during free fibula mandibular reconstruction, which improves TMJ function. TMJ dysfunctions such as clicking, inadequate incisal opening, mandibular deviation, bone resorption, and malocclusion caused by changes in condylar position may occur after surgery [6, 21]. As a result, our research examines the effects of mandibular reconstruction on condylar position, spaces, volumetric joint space, and the outcome in terms of condylar head preservation. Therefore, this is the first study that evaluates the patients as the clinical outcomes and radiographic assessment. Moreover, three-dimensional cone-beam computed tomography (CBCT) was used in this research to improve the accuracy of preoperative, postoperative, and follow-up monitoring (long-term). CBCT was also used to evaluate preoperative, immediate postoperative, and follow-up images for changes in condylar position compared to its original position through joint space measurement on the CBCT images. The authors expected that after mandibular reconstruction with condylar preservation, the condylar position would shift immediately after surgery and over time (follow up), and on the opposite side would suffer impaired function. Three-dimensional (3D) bilateral TMJs were analyzed preoperatively, postoperatively, and during follow-up with CBCT images. Parameters included the condylar position and inclination, circumferential, and volumetric joint space, using the cranial base as a reference point. The use of three-dimensional measurements describes any fastidious condylar changes that occur without deformation or superimposition. Few studies have reported the operative performance, whereas changes in condylar position have been inadequately studied. To date, analyses have been carried out using plain radiographs or CT scans, which are inadequate in analyzing the complicated condylar area and are often not investigated systematically [22, 23].

CBCT is a perfect tool for assessing craniomandibular articulation and is considered the appropriate measure for evaluating the anatomic structures leading to the best diagnosis and treatment planning [24]. Also, it allows a very detailed assessment of details of the skeletal anatomy with the advantage of avoiding superimposition and interferences with other structures compared to 2D imaging facilities [13, 25, 26]. In the current study, the assessments of postoperative changes were performed on the CBCT to obtain the most appropriate results.

All the coefficient of reliability (R) values (intra-observer) were above 0.95, which means that the measurement error for this study is negligible. Intraclass correlation coefficient (ICC) and absolute and relative technical error of measurement were between 0.972–0.999, stating the accepted reliability between intraobserver times.

One of the most common complications of mandibular reconstruction is condylar displacement after surgery. It can cause relapse and symptoms of temporomandibular joint dysfunction. According to previous studies, the condyle displaces in different directions after mandibular surgery [27, 28]. Abnormal mandibular movement, fixation methods, segment rigidity, or masticatory muscle tension can all force the condyle to migrate out of or into the fossa. Postoperative complications can occur due to condylar displacement resulting from the mandibular reconstruction. After surgery, condylar displacement and the slope of the condylar long axis might have a significant effect [29].

The condyle position to the midsagittal plane on the tumor side was significantly higher than that on the non-tumor side. Regarding the condylar position, on the tumor side, it is located far away from the midsagittal plane (ML-CP) in T1-T3 in comparison with the non-tumor side with statistical significance (*P* < 0.004). The distance between the condyle and the horizontal plane (V-CP) in the tumor side in T2-T3 is more than the non-tumor side with statistical significance (*P* < 0.004), which was supported by the significant increase of the superior joint space. The distance between the condyle and the vertical plane (AP-CP) was approximately the same on both sides. This study of orthognathic: condylar positional changes after sagittal split osteotomy for mandibular advancement found that condylar displacements following bilateral sagittal split osteotomies for mandibular advancement have a significant correlation with the degree of mandibular advancement. However, concomitant maxillary osteotomies have no influence on the condylar positional changes [30]. The anterior joint space on the tumor side was higher than on the non-tumor side. This result is consistent with findings in previous studies [13, 31, 32]. These findings might be due to the flattening of the arch width along with the laterally positioned tumor side and the CBCT accurate localization of the condyle points in the three planes, which produce more precise details [33, 34].

Among the three time point measurements in T1-T2, T1-T3, and T2-T3. The present results indicated that condyle on the tumor side move anteroinferiorly immediately after the surgery (T1-T2), which is in line with findings in previous studies [11, 35, 36], but in contrast with findings of other previous studies that show condyle move posteroinferiorly immediately after surgery (T1-T2). This, in turn, is also in comparison with the findings of other prior studies and tends to move anterosuperiorly during follow-up (T2-T3), which is the same as the findings of previous studies [11, 35]. Also, the condyle in the non-tumor side moves immediately after surgery (T1-T2) and tends to counite and move anteriorly during follow-up (T2-T3), which is in contrast with the findings of previous studies [11]. The superior joint space was also more statistically significant on the tumor side than on the non-tumor side. These variances indicate that the condyles were changed considerably from the original preoperative position.

Multiple factors may affect the various outcomes observed in this study. The occlusion and neuromuscular environment of patients requiring a recovery time is influenced by reconstruction surgery. Moreover, even though masticatory muscles were reattached and balanced in new places during recovery, overexertion and muscular stretching were insufficient to maintain the condyles in the preoperative position [6].

In the literature TMD, malocclusion, and involved muscle activity have all been related using the Helkimo index [37, 38]. So, the current study is the first to use CBCT and the Helkimo index to evaluate the accuracy of condyle position morphology in unilateral mandibular reconstruction by fibula flab. Moreover, the evaluation systems, such as the Helkimo index, can be used to determine TMJ functional impairment [39]. The Helkimo index was also used to examine movement, joint function after mandibular reconstruction, mandibular condyle fractures, pain, musculature, TMD, and malocclusion, which provides a rapid, objective assessment that could be useful at various stages of therapy [14]. Regarding the Helkimo index correlation with condyle position and joint spaces, there was no significant relationship between the Helkimo index result and variables at T3 (*P* > 0.05), which indicates that the condyle position has a good adaptive position.

## Conclusion

The condyle positions on the tumor side changed noticeably and overtime after mandibular reconstruction with condylar preservation, and the non-tumor side was affected. The condyle on the tumor side was displaced far from the horizontal plane in T1-T3 compared to the non-tumor side, with statistically significant differences. This means the condyles were permanently moved from their original preoperative position, even though they were moved during surgery.

Finally, the volumetric joint space on the tumor side is wider than on the non-tumor side; this change shows statistically significant differences on the tumor side at T1-T2 and T1-T3 *P* < 0.05. This change was transiently changed in which the space returned to the original at the last follow-up period.

## Supplementary Information

**Additional file 1.**

## Data Availability

The datasets used and/or analyzed during the current study are available from the corresponding author upon reasonable request.
