# Supplementary material for: Three dimensional condylar positional and morphological changes following mandibular reconstruction based on CBCT analysis: a prospective study
Source: Head Face Med. 2023 Feb 7;19:3. doi: 10.1186/s13005-023-00347-4 (PMC9903492; doi:10.1186/s13005-023-00347-4)
Supplement: Supplementary file 1 — Additional file 1. [file 13005_2023_347_MOESM1_ESM.pdf]

兰州大学口腔医学院临床科学研究伦理委员会审查批准函

编号：LZUKQ-2019-007　　　　申请日期：2019 年 9 月 1 日

项目名称	Three dimensional Positional and Morphological Changes of condylar Following Mandibular Reconstruction: CBCT analyzed prospective Study's analysis.<br>下颌骨重建后髁突的三维位置和形态变化：CBCT 前瞻性研究分析。				
项目类别	临床研究				
承担科室	颌面外科	负责人	谢富强	项目状态	在研
出席会议人数			弃权或废票		
同　意		修改后同意		不同意	终止或暂停
9 票		0 票		0 票	0 票

声明：我将自觉接受兰州大学口腔医学院伦理委员会的监督与检查，并保证上述材料客观可靠。

项目负责人签（章）：谢富强 [illegible]

项目执行人签（章）：谢富强 [illegible]

2019 年 9 月 1 日

1 伦理委员会对项目的审查决定如下（在意见后面打勾）。

同意 ✓　　修改后同意　　不同意　　终止或暂停

2 经伦理委员会审查认为该项目符合伦理要求。

3 该项目实施过程中将接受伦理委员会的持续审查。

主任委员签名：[illegible]

伦理委员会盖章：

日期：　　年　　月　　日

1 请严格按照院伦理委员会通过的方案执行。

2 研究过程中，对研究方案及知情同意书等文件所做的任何修改，均需得到院伦理委员会的审查同意后方可实施。

3 研究中发生的严重不良事件及意外不良事件均需及时通报伦理委员会，伦理委员会有权限依据评估结果做出新的决定。

4 研究过半时，需向伦理委员会提交中期报告。

5 对于违反伦理委员会通过方案者，委员会有权终止该研究。
